# AMPK Inhibits ULK1-Dependent Autophagosome Formation and Lysosomal Acidification via Distinct Mechanisms

**DOI:** 10.1128/MCB.00023-18

**Published:** 2018-04-30

**Authors:** Chinwendu Nwadike, Leon E. Williamson, Laura E. Gallagher, Jun-Lin Guan, Edmond Y. W. Chan

**Affiliations:** a Strathclyde Institute for Pharmacy and Biomedical Sciences. University of Strathclyde, Glasgow, Scotland; b Department of Cancer Biology, University of Cincinnati College of Medicine, Cincinnati, Ohio, USA; c Department of Biomedical and Molecular Sciences, School of Medicine, Queen's University, Kingston, Ontario, Canada; d Department of Pathology and Molecular Medicine, Kingston General Health Research Institute, Kingston, Ontario, Canada

**Keywords:** autophagy, amino acid starvation, glucose starvation, ULK1, MTORC1, AMPK, leucine, glutamine, arginine, lysosome acidification

## Abstract

Autophagy maintains metabolism in response to starvation, but each nutrient is sensed distinctly. Amino acid deficiency suppresses mechanistic target of rapamycin complex 1 (MTORC1), while glucose deficiency promotes AMP-activated protein kinase (AMPK). The MTORC1 and AMPK signaling pathways converge onto the ULK1/2 autophagy initiation complex. Here, we show that amino acid starvation promoted formation of ULK1- and sequestosome 1/p62-positive early autophagosomes. Autophagosome initiation was controlled by MTORC1 sensing glutamine, leucine, and arginine levels together. In contrast, glucose starvation promoted AMPK activity, phosphorylation of ULK1 Ser555, and LC3-II accumulation, but with dynamics consistent with a block in autophagy flux. We studied the flux pathway and found that starvation of amino acid but not of glucose activated lysosomal acidification, which occurred independently of autophagy and ULK1. In addition to lack of activation, glucose starvation inhibited the ability of amino acid starvation to activate both autophagosome formation and the lysosome. Activation of AMPK and phosphorylation of ULK1 were determined to specifically inhibit autophagosome formation. AMPK activation also was sufficient to prevent lysosome acidification. These results indicate concerted but distinct AMPK-dependent mechanisms to suppress early and late phases of autophagy.

## INTRODUCTION

During macroautophagy (here called autophagy), cellular components are sequestered into double-bilayer membrane vesicles termed autophagosomes. Autophagosomes are transported to lysosomes, followed by organellar fusion to allow content degradation and recycling of metabolic building blocks for cell viability (1, 2). A fundamental feature of autophagy is that it is potently induced following nutrient starvation, for example, in Saccharomyces cerevisiae deprived of nitrogen (amino acids and ammonia) (3). Autophagy is widely acknowledged to be a central hub for maintaining metabolic homeostasis, which plays roles in the larger context, controlling cell fate during normal ageing and cancer cell survival (4). As such, we and others have been interested in how the mammalian ULK1/2 complex coordinates multiple nutrient-dependent signals at the top of the autophagy regulatory cascade.

In one prominent model, mechanistic target of rapamycin complex 1 (MTORC1) phosphorylates ULK1 on Ser757 (Ser758 in humans), which has the effect of disrupting interaction between ULK1 and AMP-activated protein kinase (AMPK) (5). This direct binding allows AMPK to phosphorylate ULK1 on sites Ser317 and Ser777, which stimulates ULK1 activity for autophagy. Amino acid starvation would suppress MTORC1 activity, facilitating positive autophagy regulation from AMPK. Glucose starvation would in turn activate AMPK to promote autophagy via ULK1-mediated phosphorylation of factors such as beclin1, ATG13, and FIP200 (6, 7). This single model, however, cannot account for the full complexity of autophagy, which involves other modifications of ULK1. AMPK phosphorylates ULK1 on other sites, such as Ser467, Ser555, Ser574, and Ser637 (Ser467, Ser556, Ser575, and Ser638 in humans), which may function for mitophagy in response to cell energy signals (8, 9). Other patterns of nutrient-sensitive phosphorylation on ULK1 have been reported, and the Ser637 site appears to be controlled by both MTORC1 and AMPK, highlighting interconnections not yet fully understood (10).

Autophagy induction following amino acid starvation is widely prevalent, robust, and rapid (11–13). Autophagy following glucose starvation has also been reported, but this response appears to be more complex, requiring more prolonged stress to produce effects (5, 14–19). Interestingly, the MTORC1-AMPK-ULK1 interplay model predicts that autophagy following amino acid withdrawal still requires AMPK function. Regarding this issue, the precise roles of glucose starvation and AMPK in autophagy remain controversial. Low cellular energy levels and AMPK activation were initially proposed to block autophagy, based on biochemical approaches (20, 21). Other reports have shown glucose starvation to inhibit autophagy responses (22, 23, 24).

Previously, we approached this area by studying nutrient-dependent autophagy in fibroblasts derived from ULK1/2 double-knockout (DKO) mice (11). We showed that ULK1/2 DKO clearly blocked the rapid autophagy response stimulated by amino acid starvation. In that work, we noted that prolonged (overnight) glucose starvation produced a distinct autophagy phenotype that was independent of canonical phosphatidylinositol 3-phosphate (PtdIns3P) signals. Here, we further investigated how amino acid and glucose starvation signals control autophagy. We found, in a wide range of cells, that only amino acid starvation stimulated robust bona fide autophagy degradative flux. In contrast, glucose starvation produced phenotypes resembling a reduction of flux and halted autophagy. Surprisingly, amino acid starvation and glucose starvation showed differential control of autophagy gene expression, early autophagosome formation, and activation of the lysosome. Furthermore, glucose starvation and the resulting AMPK activation could override and suppress amino acid starvation signals that normally trigger autophagy. These findings highlight the opposing mechanisms that allow MTOR and AMPK to balance the functions of both early and late stages of autophagy.

## RESULTS

### Glucose starvation fails to activate autophagy flux.

**FIG 1:**
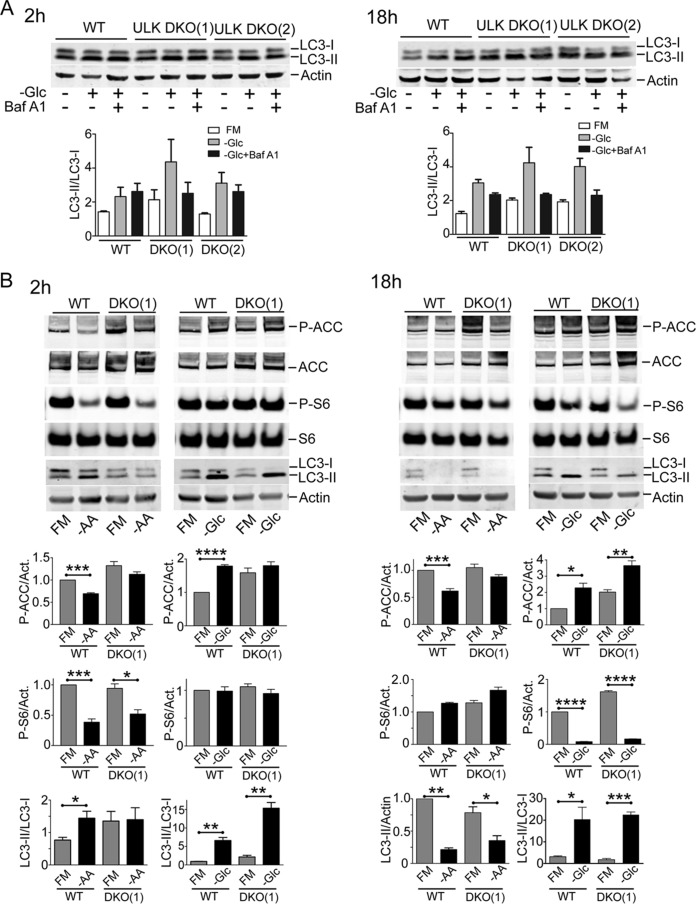
Glucose starvation does not activate autophagy flux. (A) Wild-type or ULK1/2 DKO MEF were exposed (+) or not exposed (−) to glucose starvation (−Glc), in the presence (+) or absence (−) of Baf A1, as indicated, for 2 or 18 h. Starvation conditions included 10% dialyzed FBS. FM, full-nutrient control medium. The cell lysates were analyzed for LC3B lipidation and quantified as LC3-II/LC3-I (*n* = 3 experiments; the error bars indicate standard errors of the mean [SEM]). Two different lines of ULK1/2 DKO MEF were analyzed. (B) Cells were exposed to amino acid (−AA) or glucose starvation for the indicated times. Starvation conditions included 10% dialyzed FBS. The cell lysates were analyzed for LC3B, phospho- or total ACC, and phospho- or total ribosomal S6 (S6). Quantification was based on 3 experiments; the error bars indicate SEM. *, *P* < 0.05; **, *P* < 0.01; ***, *P* < 0.001; ****, *P* < 0.0001; *t* test.

We have previously shown how amino acid starvation robustly activated autophagy in mouse embryonic fibroblasts (MEF) and how this response was fully blocked upon ULK1/2 DKO (11). Here, we explored alternate forms of nutrient stress. Surprisingly, we found that starving MEF of glucose did not strongly activate autophagy, as detected by conversion of inactive LC3-I to activated (lipid-modified) LC3-II (Fig. 1A). Glucose starvation led to only relatively small increases in LC3-II that did not further accumulate when lysosomal activity was blocked by bafilomycin (Baf) A1, which clearly contrasted with our previous observations following amino acid starvation using the same cell system (11). Glucose starvation failed to activate Baf A1-dependent LC3-II accumulation in both short (2-h) and prolonged (18-h) starvation experiments. Furthermore, the mild changes in LC3-II following glucose starvation still occurred in ULK1/2 DKO MEF lines.

We confirmed that amino acid starvation within 2 h led to clear MTORC1 suppression (S6 phosphorylation levels) and LC3 conversion (Fig. 1B). Amino acid starvation was properly sensed as MTORC1 suppression in ULK1/2 DKO MEF, although LC3 conversion was not activated. In contrast, 2 h of glucose starvation promoted AMPK activation (acetyl coenzyme A [acetyl-CoA] carboxylase [ACC] phosphorylation) in wild-type (WT) MEF but little change in MTORC1 activation. LC3-II was indeed formed following glucose starvation, but independently of ULK1/2 function. We interpret this change to represent a block in lysosomal flux, as discussed below (see Fig. 10).

For further clarification, we studied longer-term effects. MTORC1 activity recovered after overnight amino acid starvation, and cells showed dramatically low total LC3 protein levels in both WT and ULK1/2 DKO MEF (Fig. 1B). In contrast, overnight glucose starvation led to mild AMPK activation and MTORC1 suppression in both WT and ULK1/2 DKO cells. As such, the cells appear to shut down MTORC1, albeit slowly, following glucose starvation, which may reflect the AMPK- or RagA-dependent glucose-sensing mechanisms previously reported (25, 26). LC3-II accumulated similarly in both cell types following prolonged glucose starvation. Together, these data suggest that only amino acid starvation activates a ULK1/2-dependent immediate autophagy response. Prolonged amino acid starvation activates a further, ULK1/2-independent degradative pathway that leads to LC3 clearance, ultimately leading to MTORC1 reactivation (27). As we show below (see Fig. 10), this likely represents amino acid starvation-based activation of the lysosome. Glucose starvation failed to robustly activate immediate or long-term autophagy degradative flux. Since MEF generally showed a strong requirement for survival growth factors, particularly during prolonged starvation, these incubation mixtures all contained dialyzed serum, and only effects from glucose or amino acid starvation were studied.

**FIG 2:**
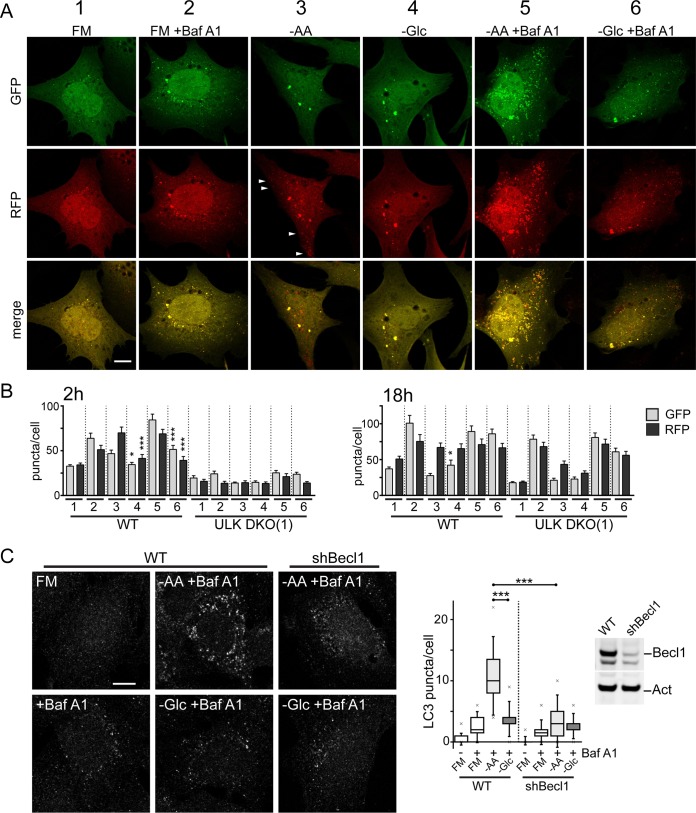
Amino acid starvation activates rapid beclin1-dependent autophagy flux. (A) Wild-type MEF expressing mRFP-EGFP-LC3B were exposed to FM or amino acid (−AA) or glucose (−Glc) starvation in the presence of Baf A1, as indicated, for 2 h. Starvation conditions included 10% dialyzed FBS. Autophagy membranes visible in the GFP and RFP channels are shown. The arrowheads show RFP^+^-only (i.e., GFP-quenched, late) autophagosomes. Scale bar, 10 μm. (B) GFP- and RFP-positive autophagy membranes were quantified in wild-type or ULK1/2 DKO MEF expressing mRFP-EGFP-LC3B following starvation conditions 1 to 6 (as in panel A) for 2 or 18 h. Quantification was done using 30 to 40 cells from three 2-h experiments or 20 to 32 cells from two 18-h experiment; the error bars indicate SEM. *, *P* < 0.05; ***, *P* < 0.001; unpaired *t* test comparing conditions 3 versus 4 and 5 versus 6. (C) MEF (wild type or with stable beclin1 knockdown [shBecl1]) were starved as indicated with Baf A1 for 2 h. Starvation conditions included 10% dialyzed FBS. Endogenous LC3B-positive autophagy membranes were detected and quantified in 120 cells (from 2 experiments). Beclin1 knockdown efficiency was confirmed. Boxes show the 25th and 75th percentiles and means, and the whiskers show standard deviations. ×, 1st and 99th percentiles; ***, *P* < 0.001; ANOVA with Tukey's posttest.

The complex regulatory effects of amino acid versus glucose starvation required further clarification, so we tested autophagy membrane flux in MEF expressing tandem monomeric red fluorescent protein (mRFP)-green fluorescent protein (GFP)-tagged LC3 (28) (Fig. 2A). We confirmed that 2 h of addition of Baf A1 alone under full-nutrient conditions deacidified and revealed all the basal autophagosomes in resting cells. Amino acid starvation of MEF for 2 h produced mild increases in GFP-detectable (i.e., early) and RFP-detectable (i.e., total) autophagosomes. Notably, amino acid starvation produced RFP^+^-only membranes, which represent autophagosomes that acidify and mature into late degradative compartments. Glucose starvation also led to mild changes in GFP- and RFP-visible membranes, but the level of response was significantly less than that of amino acid starvation upon quantification of cell populations (Fig. 2B) and did not produce RFP^+^-only autophagosomes arising from flux.

In the presence of Baf A1, amino acid starvation for 2 h led to strong accumulation of autophagosomes (visible with GFP and RFP due to global deacidification). In contrast, glucose starvation plus Baf A1 produced a markedly lower level of autophagosome formation. Moreover, the amino acid starvation and Baf A1 responses at 2 h were clearly blocked upon ULK1/2 DKO. When the experiment was performed following 18-h starvations, we detected a distinct pattern (Fig. 2B). Baf A1 alone or both starvation conditions (plus Baf A1) all led to similar accumulation of GFP^+^ and RFP^+^ membranes. Furthermore, the accumulations took place similarly, even with ULK1/2 DKO. These results suggested that only amino acid starvation (in the short term) stimulated canonical autophagy flux and that this response was strictly dependent on the ULK1/2 complex. Upon prolonged (e.g., overnight) starvation, other, lower-rate autophagy-related processes become more apparent, but they did not display differential sensitivity to amino acid versus glucose starvation and were ULK1/2 independent.

We further validated our findings using another imaging approach, detecting endogenous LC3-positive autophagosomes in starved WT MEF (Fig. 2C). Baf A1 alone (under control conditions) led to only small accumulation of basally forming autophagosomes. LC3-positive autophagosome formation was strongly promoted by amino acid, but not glucose, starvation. Since activated ULK1/2 promotes autophagy by phosphorylating downstream signaling partners, such as beclin1 (6), we further confirmed that the short-term amino acid-dependent autophagy response was blocked upon Beclin1 silencing.

### Differential nutrient responses in cancer cells.

**FIG 3:**
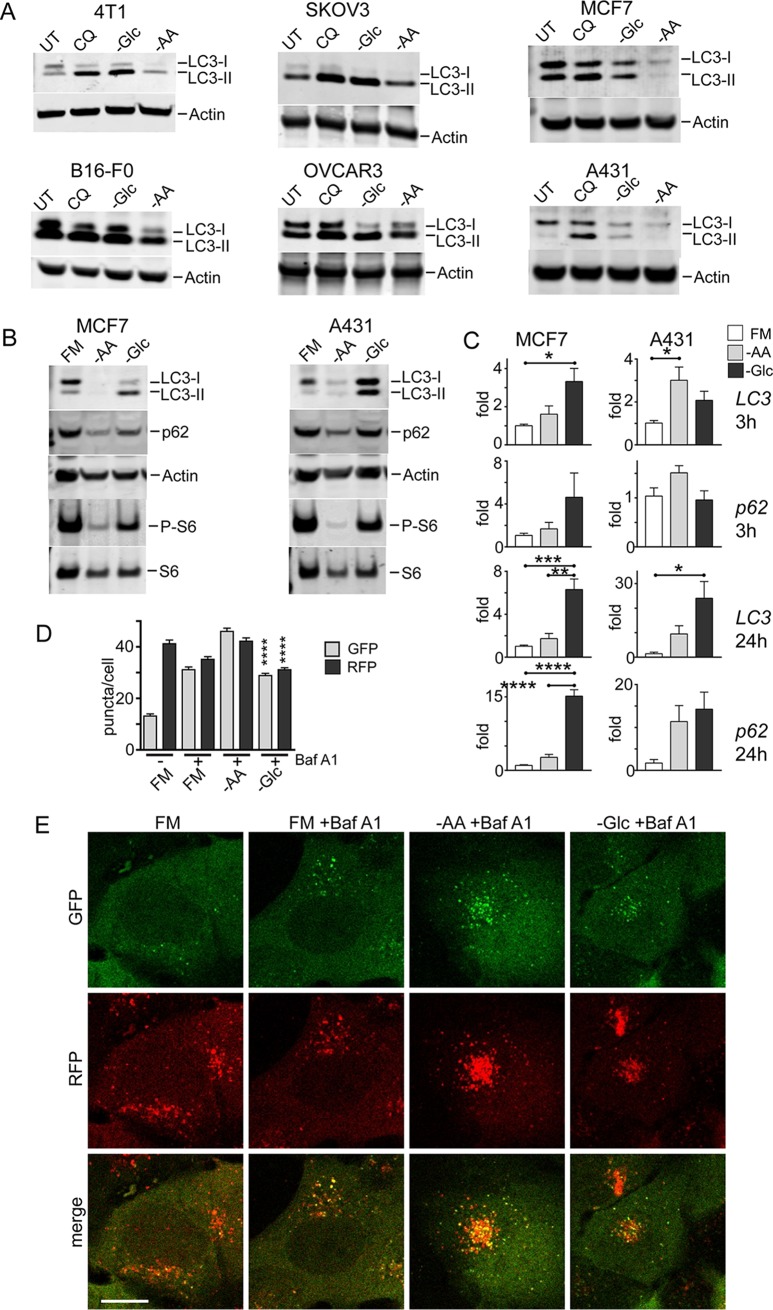
Lack of autophagy flux following glucose starvation is conserved in cancer cells. (A) The indicated cell types were incubated with glucose or amino acid starvation for 3 h. As a control, CQ (25 μM) was added to cells under full-nutrient conditions. UT, untreated. (B) MCF7 or A431 cells were treated with prolonged starvation (24 h) as indicated. Starvation conditions included 10% dialyzed FBS. Cell lysates were analyzed for LC3B, sequestosome 1/p62 protein levels, and S6 protein phosphorylation. The data are representative of the results of 3 experiments. (C) MCF7 or A431 cells were starved as indicated for 3 or 24 h. Transcript levels for *LC3B* and *p62* were quantified (expressed as fold change normalized to *GAPDH*; *n* = 3; the error bars indicate SEM). *, *P* < 0.05; ***, *P* < 0.001; ****, *P* < 0.0001; ANOVA with Tukey's posttest. (D and E) A431 cells stably expressing mCherry-EGFP-LC3B were starved as indicated with Baf A1 for 2 h. Starvation conditions included 10% dialyzed FBS. GFP- and RFP-positive autophagy membranes were quantified in 120 cells from 3 experiments; the error bars indicate SEM. ****, *P* < 0.0001; unpaired *t* test comparing –AA and –Glc conditions. Scale bar, 10 μm.

The failure of glucose starvation to activate autophagy was puzzling, so we investigated if this trend was conserved, particularly in cancer cells that exhibit high glucose catabolism. We found, in a range of breast, ovarian, and melanoma cancer cell lines, that glucose starvation generally led to LC3-II accumulation, similar to cells with lysosomal inhibition via chloroquine (CQ) (Fig. 3A). This similarity was most obvious in 4T1, SKOV3, and OVCAR3 cells but was generally displayed in the other cell types. In contrast, amino acid starvation over the same time frame led to patterns of LC3 conversion and clearance. MCF7 and A431 were further studied as representative cell models showing clear nutrient-dependent differences. In both cell types, overnight amino acid starvation led to strong flux and clearance of total LC3 and the sequestosome 1/p62 autophagy adaptor protein (Fig. 3B). In contrast, overnight glucose starvation did not produce strong LC3 and p62 degradation.

These results in cancer cells support our model for differential responses to amino acid versus glucose starvation. Since changes in levels of LC3 or p62 protein can arise from both autophagy and gene expression mechanisms, particularly in prolonged starvation (29), we further tested *LC3B* and *p62/SQSTM1* transcript levels (Fig. 3C). Under short-term starvation, only glucose (and not amino acid) starvation led to mild *LC3B* and *p62* upregulation in MCF7 cells. A431 displayed a mild but distinct nutrient-dependent response. Under prolonged starvation, there was clearer upregulation of LC3B and p62, particularly upon glucose starvation. The nutrient-dependent difference was especially apparent in MCF7 cells. These data suggest that in the short term, amino acid starvation activates autophagy flux, leading to loss of LC3 and p62 proteins (although there is no gene downregulation). Under prolonged time frames, amino acid starvation produces some upregulation, but LC3 and p62 degradation rates appear to overwhelm this response. In contrast, prolonged glucose starvation does not produce degradative flux and further stimulates *LC3* and *p62* upregulation. Lastly, we confirmed the differential autophagy flux by imaging A431 cells expressing mRFP-GFP-LC3 (Fig. 3D and E). Similar profiles were observed, with Baf A1 (alone under control conditions) revealing basal autophagosome levels. In the short term only amino acid, but not glucose, starvation promoted strong autophagosome formation.

### Amino acid starvation activates autophagy flux.

**FIG 4:**
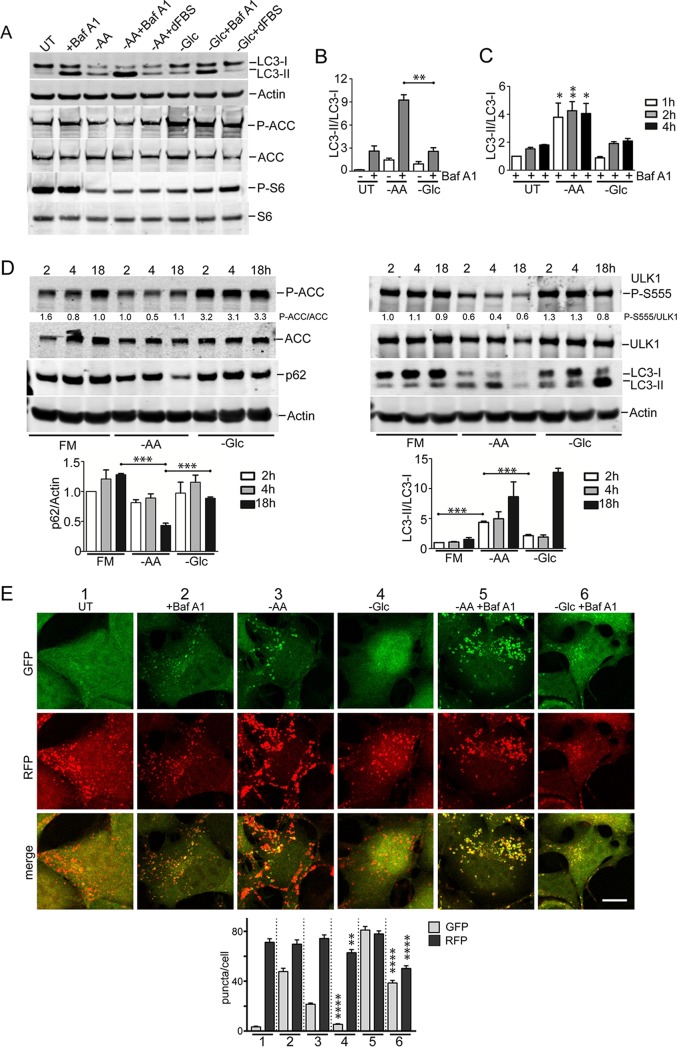
Amino acid starvation triggers dephosphorylation of ULK1 serine 555 and autophagy. (A) HEK293A cells were incubated under amino acid or glucose starvation conditions for 2 h. Where indicated, bafilomycin A1 or 10% dialyzed FBS was added. Immunoblotting detected LC3B lipidation, P-ACC, and P-S6 levels in lysates. (B) Quantification of LC3B lipidation ratios was based on 3 experiments; the error bars indicate SEM. **, *P* < 0.01; unpaired *t* test. (C) HEK293A cells were starved of amino acid or glucose for the indicated times. All conditions were with bafilomycin A1. Quantification was based on 3 experiments; the error bars indicate SEM. *, *P* < 0.05; **, *P* < 0.01; ANOVA with Tukey's posttest comparing UT and –AA conditions. (D) HEK293A cells were incubated under amino acid or glucose starvation for the indicated times. Starvation conditions included 10% dialyzed FBS. Parallel wells of control cells were replenished with full-nutrient medium at the start of incubation. The cell lysates were analyzed for ULK1 serine 555 phosphorylation, P-ACC, LC3B, and p62 levels. Representative quantification is shown below the phosphoblots. Quantification of p62 degradation and LC3B ratios was based on 3 experiments; the error bars indicate SEM. ***, *P* < 0.001; ANOVA with Tukey's posttest. (E) HEK293A stably expressing mCherry-EGFP-LC3B were incubated under the indicated starvation conditions (1 to 6) for 2 h. Starvation conditions included 0.1% dialyzed FBS. **, *P* < 0.01; ****, *P* < 0.0001; unpaired *t* test comparing conditions 3 versus 4 and 5 versus 6. Scale bar, 10 μm.

We further investigated the nutrient-dependent regulation of autophagy using HEK293 cells, which we previously used extensively to study ULK1 signaling (30, 31). HEK293 cells possess a high rate of basal autophagy, even under full nutrient conditions, as shown via clear LC3-II accumulation following lysosomal block with Baf A1 (Fig. 4A, lane 2). Amino acid (and serum) starvation led to increased LC3-II (Fig. 4A, lane 1 versus 3), and this effect was further apparent when Baf A1 was used to block autophagic flux (Fig. 4A, lane 4). Addition of dialyzed serum did not alter LC3-II accumulation (Fig. 4A, lane 3 versus 5), suggesting that the cells were primarily sensing amino acid withdrawal. Amino acid starvation (with or without serum) strongly inhibited MTORC1. As observed (see above) in multiple cell types, glucose (and serum) starvation led to some LC3-II formation, but it was not as robust as with amino acid starvation (Fig. 4A, lane 3 versus 6). Furthermore, glucose starvation (Fig. 4A, +Baf A1) did not produce more LC3-II than Baf A1 treatment alone (also shown by quantitation [Fig. 4B]). Glucose (and serum) starvation led to AMPK activation and also MTORC1 suppression. However, supplementation with dialyzed serum under glucose starvation restored MTORC1 activity and prevented LC3-II generation (Fig. 4A, lane 6 versus 8). Therefore, starvation of glucose alone produces minimal autophagy responses, consistent with our other data. Serum starvation can activate autophagy to a certain extent, but its contribution is weak when directly compared with amino acid starvation.

We and others have noted glucose-dependent autophagy effects, particularly following prolonged starvation (5, 11, 14, 19); therefore, here, we investigated details in the time course of nutrient sensing. In HEK293 cells, inhibition of the lysosome led to a gradual accumulation of LC3-II over 1 to 4 h (Fig. 4C). Further amino acid starvation led to rapid formation of significantly higher levels of LC3-II. Glucose starvation failed to activate LC3-II formation above the low level caused by a lysosomal block alone.

In these prolonged experiments, we noted mild LC3 and ACC accumulation upon overnight incubation of control cells maintained with full nutrients (Fig. 4D). In contrast, amino acid starvation triggered LC3 conversion within 2 h, becoming clearer by 4 h, which is consistent with other data. By 18 h of amino acid starvation, total LC3 levels markedly decreased due to continued degradation (also reflected by p62 reduction). AMPK activity remained low during amino acid starvation. The parallel time course from glucose starvation was distinct, with less LC3 conversion and clearance, no p62 loss, and rapid AMPK activation.

Although different cell types display slightly varying responses, nutrient-dependent autophagy flux, as detected by RFP-GFP-LC3, has been consistent. In HEK293 cells (Fig. 4E), 2 h of Baf A1 treatment alone showed the basal autophagosome levels. Amino acid starvation produced significantly more GFP^+^ and RFP^+^ autophagosomes under conditions of both Baf A1 presence and absence.

### Amino acid starvation stimulates autophagosome formation in a glucose-dependent manner.

**FIG 5:**
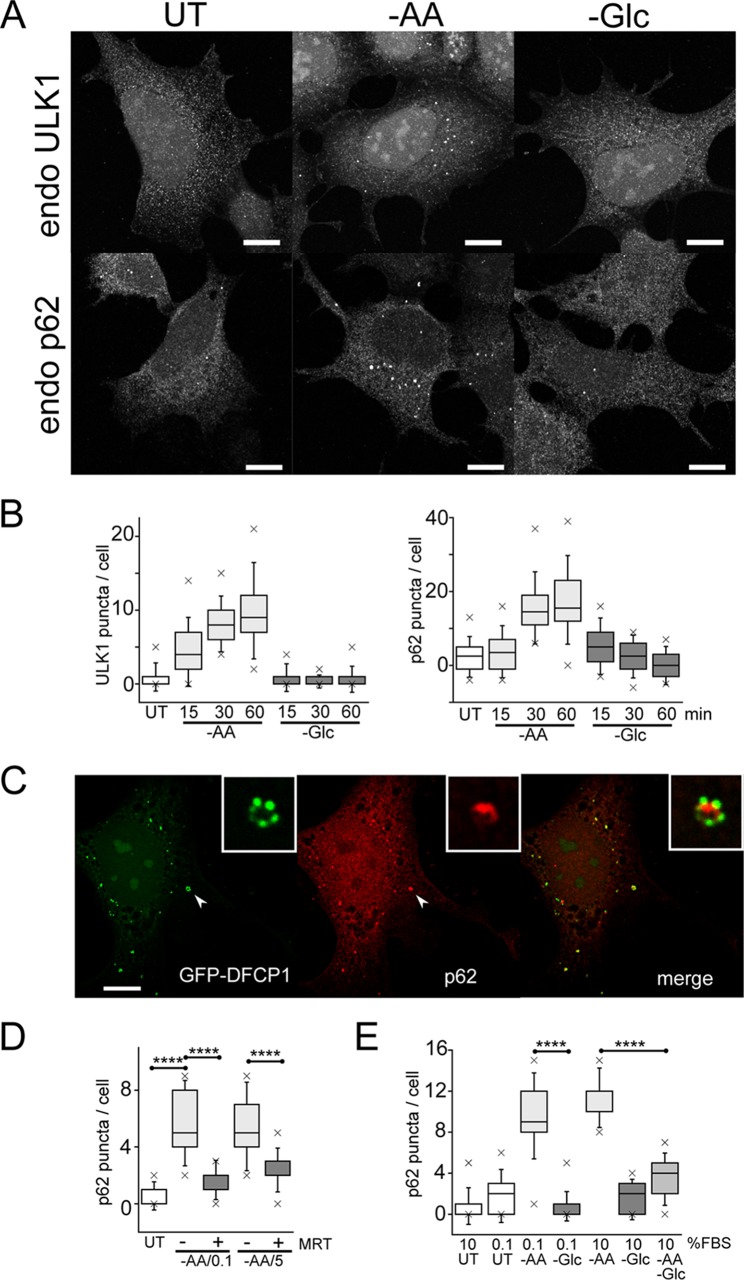
Glucose starvation inhibits autophagosome formation. (A) HEK293A cells were incubated under amino acid or glucose starvation for 2 h. Starvation conditions included 0.1% dialyzed FBS. Cells were stained for endogenous ULK1- or p62-labeled autophagosomes. Scale bars, 10 μm. (B) HEK293A cells were starved as for panel A for the times indicated, and ULK1 and p62 puncta/cell were quantified. Each box represents 38 cells, representative of the results of 2 experiments. (C) Wild-type MEF expressing GFP-DFCP1 were starved of amino acids as for panel A for 2 h. The cells were stained for endogenous p62 puncta. The arrowheads point to the autophagosome shown enlarged in the insets. Scale bar, 10 μm. (D) HEK293A cells were starved of amino acids for 2 h. Starvation conditions included 0.1 or 5% dialyzed FBS or addition of MRT68921 (10 μM), as indicated. Each box represents 40 cells, representative of the results of 2 experiments. (E) HEK293A cells were starved of amino acids, glucose, or both together for 2 h. Dialyzed serum concentrations were also varied during starvation, as indicated. Each box represents 40 to 50 cells, representative of the results of 2 experiments. In box-and-whisker plots, the boxes show the 25th and 75th percentiles and means, and the whiskers show standard deviations. ×, 1st and 99th percentiles; ****, *P* < 0.0001; ANOVA with Tukey's posttest.

As we established the nutrient-specific effects on autophagy flux, we further studied regulation of autophagosome formation. We first investigated early autophagy factor recruitment by detecting the ULK1 complex, which translocates to initial endoplasmic reticulum (ER)-associated assembly sites. We and others have previously reported clear formation of membranes using exogenous tagged ULK1 complex members (30, 32). Here, we observed that cells maintained under full nutrients contained virtually no membranes staining for endogenous ULK1 (Fig. 5A). Amino acid starvation stimulated assembly of ULK1-positive membranes within 15 min, which further increased over time (Fig. 5B). In contrast, glucose starvation did not stimulate ULK1-positive puncta.

To study a later stage of autophagosome assembly, we detected endogenous patterns of p62/sequestosome 1, which is recruited to form autophagy membranes via both LC3-dependent and -independent mechanisms (33, 34). Amino acid starvation induced p62-positive membranes in a robust, rapid, and time-dependent manner (Fig. 5A and B). In contrast, glucose starvation did not induce p62 membranes. To confirm the p62 staining, we detected colocalization with GFP-DFCP1. In WT MEF, amino acid starvation stimulated the formation of DFCP1-positive autophagosomes with concentrated patches of PtdIns3P (Fig. 5C). p62 puncta colocalized with GFP-DFCP1, intermingling with the patches of PtdIns3P on autophagosomes, suggestive of cargo recruitment. The amino acid-dependent induction of p62 puncta could be blocked with the ULK1 inhibitor MRT68921 (35) (Fig. 5D). Therefore, the data suggest that amino acid starvation rapidly promotes ULK1 activation and translocation, thereby promoting downstream p62 recruitment and autophagosome formation.

**FIG 6:**
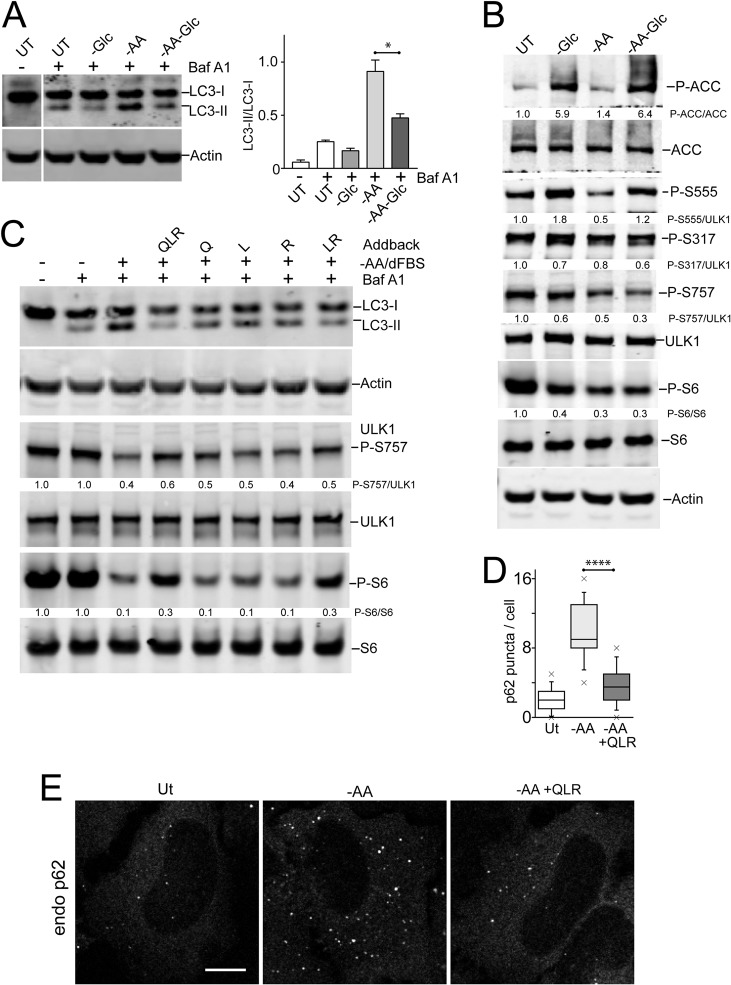
Differential effects of glucose and amino acids (glutamine, leucine, and arginine) on autophagy signaling. (A) HEK293A cells were starved of amino acids, glucose, or both together for 2 h. Starvation conditions included 10% dialyzed FBS. Bafilomycin A1 was included as indicated. The cell lysates were analyzed for LC3B lipidation and quantified based on 3 experiments; the error bars indicate SEM. *, *P* < 0.05; unpaired *t* test. (B) HEK293A cells were starved as for panel A and analyzed for P-ACC, P-S6, and phosphorylation at the indicated ULK1 sites. Representative quantification is shown below the phosphoblots. (C) HEK293A cells were starved of amino acids (in the presence of 10% dialyzed FBS). Where indicated, starvation media contained bafilomycin A1 with the amino acids glutamine (Q), leucine (L), and/or arginine (R) added back. The data are representative of the results of 3 experiments. (D and E) HeLa cells were starved of amino acids (in the presence of 5% dialyzed FBS), with amino acids added back as indicated. Endogenous p62 puncta were analyzed. Scale bar, 10 μm. (D) Each plot represents 40 cells, representative of the results of 2 experiments. Boxes show the 25th and 75th percentiles and means, and the whiskers show standard deviations. ×, 1st and 99th percentiles; ****, *P* < 0.0001; unpaired *t* test.

We further explored nutrient dependency during autophagosome formation using p62 puncta as a readout. We and others have long used Earle's balanced salt solution (EBSS) as a standard autophagy starvation medium (11, 30, 31). Notably, EBSS lacks both amino acids and serum. During the course of this study, we further clarified that serum starvation itself can mildly stimulate autophagy by suppressing MTORC1 activity (Fig. 5A). In HEK293 cells, we also observed that serum deprivation led to cytoskeletal changes but determined that supplementation with trace amounts of serum (0.1%) maintained cell morphology and prevented cell detachment. We confirmed that serum deprivation alone (from 10% to 0.1%) only mildly induces formation of p62 puncta (Fig. 5E). In comparison, further starvation of amino acid (but not of glucose) strongly induced p62 puncta. This difference between amino acid starvation and glucose starvation was observed even when starvation was performed in the context of 10% dialyzed serum. Importantly, while amino acid starvation promoted p62 membranes, further removal of glucose (double starvation) significantly blocked the formation of p62 puncta. We further confirmed biochemically that glucose starvation inhibited the amino acid starvation signal from promoting LC3 lipidation (Fig. 6A). Therefore, glucose starvation blocked the otherwise strong induction by amino acid starvation of autophagosome formation.

Since amino acid starvation and glucose starvation had opposite effects on autophagy, we asked how these nutrients were being sensed. As expected, glucose starvation led to AMPK activation and strong phosphorylation of ACC (Fig. 6B). This condition also increased phosphorylation of ULK1-Ser555. In contrast, amino acid starvation led to suppression of MTORC1 signaling without strong AMPK activation (P-ACC). Under amino acid starvation, we observed, as expected, decreased phosphorylation on ULK1-Ser757 but also decreased phospho-ULK1-Ser555. Interestingly, double starvation of both amino acid and glucose led to MTORC1 inhibition, together with AMPK activation, restoring ULK1-Ser555 phosphorylation. The other AMPK-regulated site, ULK1-Ser317 (5), showed generally steady levels throughout these starvation conditions. Altogether, these data show that maximal autophagy activation is associated with dephosphorylation on both the ULK1-Ser555 and -Ser757 sites.

### Glutamine, leucine, and arginine activate MTORC1 to inhibit autophagosome formation.

All the above-described data highlighted the primacy of amino acids for autophagy regulation. Certain amino acids, such as glutamine, leucine, and arginine play key regulatory roles by interacting with specific cellular nutrient sensors to activate MTORC1 (36–39). Here, we further tested the role of each of these key regulatory amino acids. In control samples, HEK293 cells starved of all 20 amino acids showed MTORC1 suppression and LC3 lipidation (Fig. 6C). However, adding back glutamine, leucine, and arginine to the starvation mixture prevented both MTORC1 inactivation and LC3 lipidation. Interestingly, adding back glutamine, leucine, or arginine singly did not have a strong reversal effect compared to all three regulatory amino acids added together. Adding back a combination of leucine and arginine did reduce autophagy, but not as clearly as the three combined regulatory amino acids. Adding back these regulatory amino acids required the presence of serum to activate MTORC1 and suppress autophagy (data not shown), revealing involvement of the growth factor-TSC1/2-Rheb pathway in combination with amino acids for MTORC1 activation.

**FIG 7:**
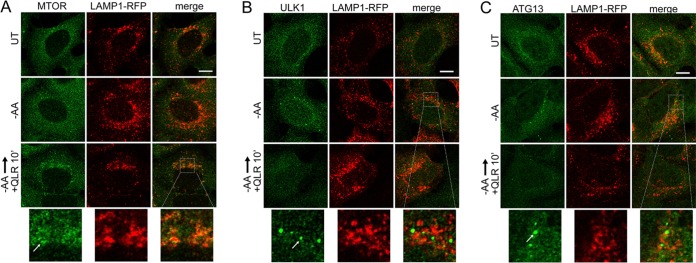
Glutamine, leucine, and arginine activate MTORC1 and inhibit ULK1 complex translocation to autophagosome assembly sites. HEK293A cells stably expressing LAMP1-mRFP were starved of amino acids for 2 h or starved for 110 min, followed by 10 min resupplementation with glutamine, leucine, and arginine (in the presence of 5% dialyzed FBS). The fixed cells were stained for endogenous MTOR (A), ULK1 (B), or ATG13 (C). The arrows in the enlarged insets indicate MTOR localized on lysosomal membranes in response to glutamine, leucine, and arginine (A) and localization of the ULK1 complex on autophagosome assembly sites juxtaposed to lysosomal membranes in response to amino acid starvation (B and C). Scale bars, 10 μm.

We confirmed that adding back glutamine, leucine, and arginine to the amino acid deprivation medium also suppressed formation of p62-positive autophagosomes (Fig. 6D and E). To further test the abilities of these 3 regulatory amino acids to control autophagy, we studied membrane translocation. We confirmed that adding back glutamine, leucine, and arginine (in the presence of serum) stimulated, within 10 min, the translocation of MTORC1 onto lysosomal compartments (Fig. 7A). To study the regulation of initiation, we monitored ULK1/2 complex localization. We confirmed the translocation of endogenous ULK1 onto membrane puncta within ∼2 h (Fig. 7B). Sites of initiation, likely connected to the ER, were generally juxtaposed but not colocalized to the lysosomal compartments. Adding back glutamine, leucine, and arginine caused ULK1 translocation of membrane puncta within 10 min, suggestive of rapid inactivation by the addition of regulatory amino acids. Staining for endogenous ATG13 showed identical results (Fig. 7C).

### AMPK phosphorylates and inhibits ULK1.

**FIG 8:**
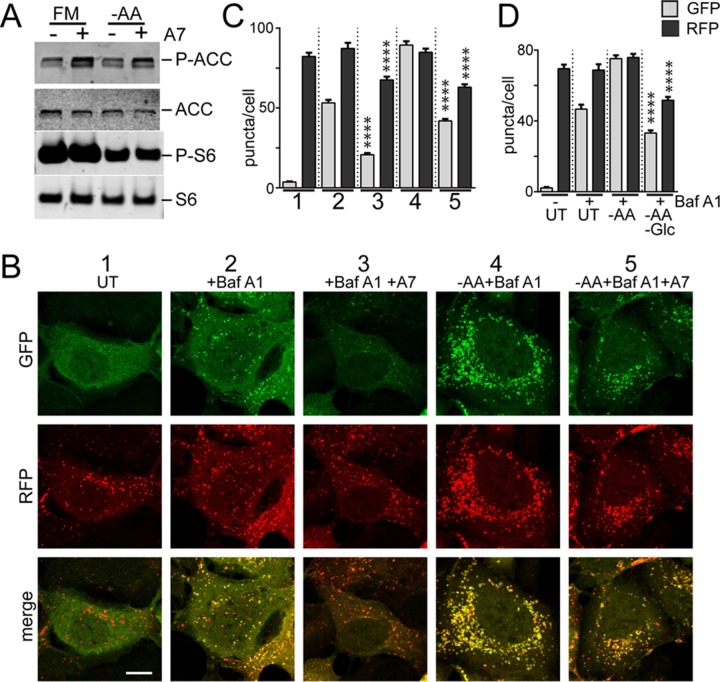
Inhibition of autophagosome formation by AMPK. (A) HEK293A cells were starved of amino acids in the presence or absence of A769662 (50 μM) for 2 h. Starvation conditions included 5% dialyzed FBS. The cell lysates were analyzed for phosphorylation of ACC and S6. (B) HEK293A cells stably expressing mCherry-EGFP-LC3B were incubated under starvation conditions (1 to 5) for 2 h. Starvation conditions included 0.1% dialyzed FBS. Scale bar, 10 μm. (C) Quantification of the data in panel B. Each plot represents 135 cells from 3 experiments; the error bars indicate SEM. ****, *P* < 0.0001; unpaired *t* test comparing conditions 2 versus 3 and 4 versus 5 (for both GFP and RFP quantifications). (D) HEK/mCherry-EGFP-LC3B cells as in panel B were starved of amino acids, or amino acid and glucose together, for 2 h. Starvation conditions included 0.1% dialyzed FBS. Each plot represents 45 cells representative of 3 experiments; the error bars indicate SEM. ****, *P* < 0.0001; unpaired *t* test comparing –AA versus double-starved conditions (for both GFP and RFP quantifications).

**FIG 9:**
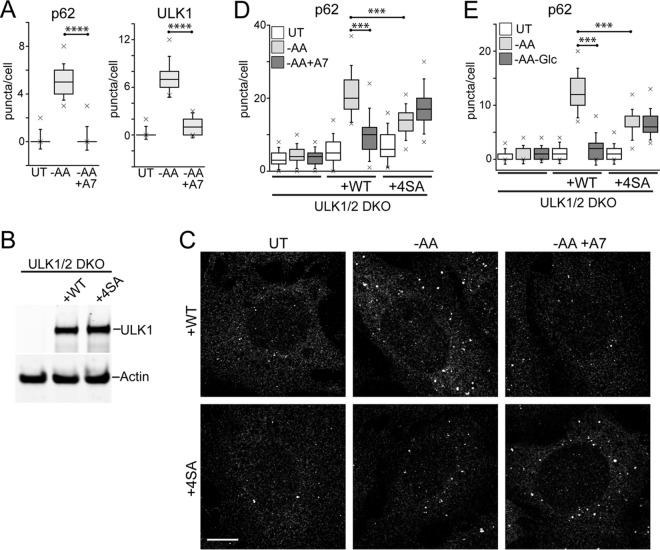
Phosphorylation of ULK1 by AMPK inhibits autophagosome formation. (A) HEK293A cells were starved of amino acids in the presence or absence of A769662 (50 μM) for 2 h. Starvation conditions included 0.1% dialyzed FBS. The fixed cells were stained for endogenous p62- or ULK1-labeled autophagosomes and quantified. Each plot represents 135 cells from 3 experiments. ****, *P* < 0.0001; unpaired *t* test. (B) ULK1/2 DKO cells were reconstituted with Myc-ULK1, wild type or 4SA (S467A, S555A, T574A, and S637A). Expression levels were confirmed by immunoblotting with anti-ULK1 antibody. (C) ULK1/2 DKO cells reconstituted with wild-type or 4SA Myc-ULK1 were starved of amino acids in the presence or absence of A769662 for 2 h. Starvation conditions included 10% dialyzed FBS. The fixed cells were stained for endogenous p62 puncta. Scale bar, 10 μm. (D) The experiment shown in panel C was quantified. Each plot represents 90 to 135 cells from 3 experiments. ***, *P* < 0.001; ANOVA with Tukey's posttest. (E) Cells as in panel C were starved of amino acids, or amino acid and glucose together, for 2 h. Starvation conditions included 10% dialyzed FBS. Each plot represents 135 cells from 3 experiments. In box-and-whisker plots, the boxes show the 25th and 75th percentiles and means, and the whiskers show standard deviations. ×, 1st and 99th percentiles; ***, *P* < 0.001; ANOVA with Tukey's posttest.

We determined that glucose starvation has the ability to suppress autophagy, even in the context of amino acid starvation cues, which promote the process. To investigate this mechanism further, we focused on AMPK, which is activated by glucose starvation. For a different approach, we used the compound A769662 to activate AMPK (40). A769662 clearly activated AMPK under both full-nutrient and amino acid starvation conditions (Fig. 8A). There were no adverse effects of A769662 on the MTORC1 pathway. We next tested the effect of AMPK activation on amino acid starvation-driven autophagy flux using the RFP-GFP-LC3 assay in HEK293 cells. The addition of A769662 significantly inhibited both basal autophagosome formation under full nutrients and autophagy stimulation by amino acid starvation (Fig. 8B and C). The effect of the AMPK activator was nearly identical to the effect of glucose withdrawal on amino acid starvation (i.e., double starvation) (Fig. 8D). The addition of A769662 similarly inhibited the ability of amino acid starvation to promote formation of puncta by ULK1 and p62 (Fig. 9A). These results support the notion that AMPK inhibits autophagy.

AMPK regulates autophagy by directly phosphorylating ULK1 on multiple sites. One set of highly conserved sites (mouse ULK1 S467, S555, T574, and S637) has been implicated in autophagy-related mitochondrial homeostasis and cell survival (9). To test the roles of these AMPK-dependent sites, we reconstituted ULK1/2 DKO MEF with either ULK1 WT or the 4SA (S467A, S555A, T574A, and S637A) mutant (Fig. 9B). We next treated the reconstituted MEF by amino acid starvation with and without AMPK activation via A769662 and monitored p62-labeled autophagosome formation (Fig. 9C and D). Reconstitution with WT-ULK1 rescued the formation of starvation-induced p62 puncta. This autophagy response in WT-ULK1-reconstituted MEF was significantly inhibited by A769662. Interestingly, MEF reconstituted with 4SA-ULK1 showed a significantly inhibited response to amino acid starvation. A pattern consistent with this was also observed when studying WT- versus 4SA-reconstituted MEF in the context of single (amino acid) versus double (amino acid-Glc) starvation (Fig. 9E). Therefore, phosphorylation of these 4 sites on ULK1 was required for AMPK to inhibit the autophagy response. However, loss of these sites also impaired the normal function of ULK1 during amino acid starvation-induced autophagy.

### AMPK and glucose starvation inhibit lysosome activity.

**FIG 10:**
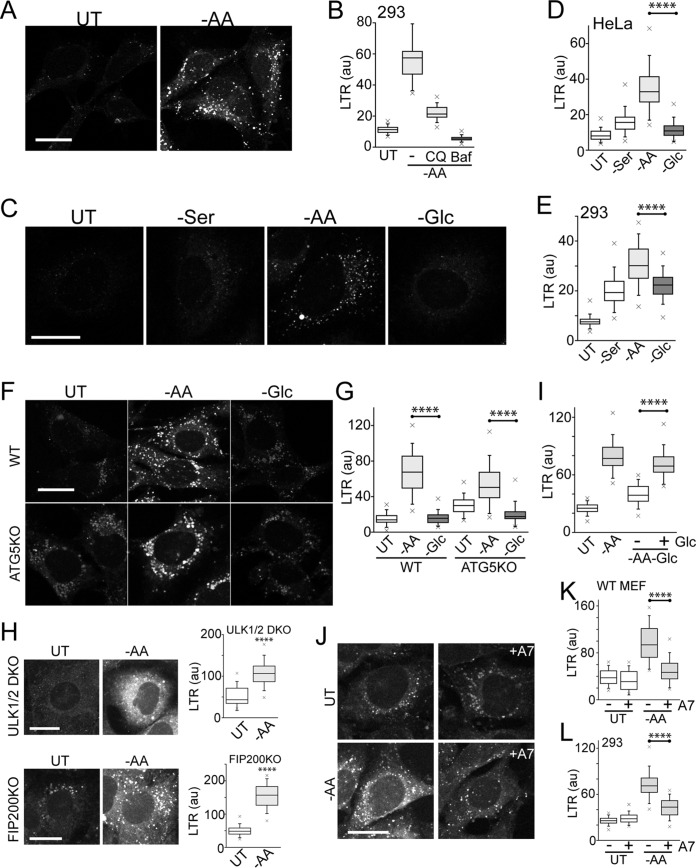
Glucose starvation and AMPK inhibit lysosomal acidification. (A and B) HEK293A cells were starved of amino acids for 2 h and stained using Lysotracker Red DND-99. Starvation conditions included 0.1% dialyzed FBS. Where indicated, starvation included CQ or bafilomycin A1. Lysotracker staining intensity per cell was quantified from confocal images (arbitrary units). Each box represents 60 cells, representative of the results of 4 experiments. (C) HeLa cells were starved of serum, amino acids, or glucose for 2 h and analyzed for Lysotracker staining. Amino acid and glucose starvation conditions included 0.1% dialyzed FBS. (D) Quantification of the data in panel C. Each box represents 50 to 60 cells, representative of the results of 4 experiments. (E) The experiment shown in panels C and D was repeated in HEK293A cells. Each box represents 50 to 60 cells, representative of the results of 4 experiments. (F and G) Wild-type or ATG5 knockout MEF were starved and analyzed as for panel C. Each box represents 40 to 50 cells, representative of the results of 5 experiments. (H) ULK1/2 DKO or FIP200 KO MEF were starved of amino acids and analyzed as for panels C and D. Each box represents 270 to 320 cells from 3 experiments. (I) HEK293A cells were starved of amino acids, or amino acid and glucose together, for 2 h as for panel C. Where indicated, the double-starvation conditions included adding back d-glucose (1 g/liter). Each box represents 60 cells, representative of the results of 4 experiments. (J to L) Wild-type MEF (or HEK293A cells [L]) were starved of amino acids in the presence or absence of A769662 (50 μM) for 2 h and analyzed as for panels C and D. Each box represents 120 cells from 2 experiments. In box-and-whisker plots, the boxes show the 25th and 75th percentiles and means, and the whiskers show standard deviations. ×, 1st and 99th percentiles; ****, *P* < 0.0001; unpaired *t* test. Scale bars, 20 μm.

Our data described above highlighted how amino acid starvation was best at activating autophagy-lysosomal flux, as seen through eventual clearance of LC3 and p62 proteins. Since MTORC1-dependent activation of lysosomal activity has been reported (41), we investigated the differential nutrient dependency on this late-stage of autophagy. We confirmed that amino acid starvation led to robust lysosomal activation in HEK293 and HeLa cells, as detected by Lysotracker Red staining for acidified vesicles (Fig. 10A). Quantification of staining indicated starvation-induced lysosomal acidification, which could be effectively quenched by treatment with the weak base CQ or, more strongly, with the vacuolar ATPase (vATPase) inhibitor Baf A1 (Fig. 10B), as seen previously (29).

In testing the different nutrients, we found that serum starvation alone led to mild acidification of the lysosome, for example, in both HeLa (Fig. 10C and D) and HEK293 (Fig. 10E) cells. However, further withdrawal of amino acids markedly led to strong lysosomal acidification. In contrast, glucose starvation did not promote acidification. We found that the preferential lysosomal activation from amino acid starvation was independent of autophagy, showing similar robust effects in WT and ATG5 KO MEF (Fig. 10F and G). Lysosomes in ATG5 KO MEF appeared swollen compared to those in WT MEF. However, even these swollen vesicles markedly increased Lysotracker staining following amino acid starvation. The ability of amino acid starvation to activate lysosomal acidification was also independent of ULK1 signaling, as seen in ULK1/2D KO and FIP200 KO MEF (Fig. 10H).

Our data described above also highlighted how glucose starvation inhibited amino acid-dependent cues that drive autophagosome formation. We tested this relationship for lysosomal acidification. Indeed, we found that while amino acid starvation stimulated lysosomes, acidification was blocked when glucose was further removed by using double-starvation medium (Fig. 10I). Adding back glucose to the double-starvation medium (to typical levels, i.e., 1 g/liter) restored acidification, indicating that cellular glucose levels promote lysosomal function. As one main effect, glucose starvation activates AMPK. To test if this pathway regulates the lysosome, we used the AMPK activator drug. Addition of A769662 had little effect on basal signals but significantly inhibited the ability of amino acid starvation to promote lysosome acidification (Fig. 10J to L). These results suggest that glucose starvation also inhibits lysosomal/late-stage autophagy via AMPK.

## DISCUSSION

Mammalian cells need to rapidly adapt when extracellular nutrients change, and a part of this metabolic homeostasis is autophagy (1, 42). Cancer cells are particularly distinct for their reconfigured metabolic profile that features high consumption of glucose and amino acids, such as glutamine (43, 44). We have long been intrigued by the mechanisms linking amino acid and glucose sensing to autophagy, particularly in cancer contexts. ULK1 appears to be a key hub receiving phosphorylation signals from MTORC1 and AMPK (5, 10). We previously investigated the different features of noncanonical ULK1/2-independent autophagy in the context of prolonged glucose starvation (11, 14). Here, upon further exploration, we found, surprisingly, that only amino acid starvation activates rapid and robust autophagy flux. In contrast, glucose starvation produced autophagy readouts more closelyresembling a lysosomal block, which was prevalent in a wide range of normal and cancer cell types.

### Amino acid and glucose differentially control autophagosome formation.

Differences in nutrient sensing could be traced to the level of autophagy initiation. Only amino acid starvation promptly promoted the translocation of ULK1 to membrane assembly sites. This is likely the key early regulatory event that allows phosphorylation of downstream substrates, such as ATG4B, ATG9, and ATG13 (35, 45–48). This is clearly a partial list of all the ULK1 substrates so far identified (as previously highlighted [7, 49]). However, this translocation critically allows the ULK1/2 complex to phosphorylate beclin1, thereby directing VPS34 activity and localized PtdIns3P generation at autophagosome assembly sites (6). Previously, we found only amino acid starvation stimulated translocation of PtdIns3P-binding WIPI-2 to autophagy membranes (11). In further agreement, here, we found that amino acid (but not glucose) starvation rapidly promoted high numbers of autophagosomes containing LC3 and p62. Consistent with established thinking, MTORC1 played the predominant role in autophagosome regulation. Readdition of the key regulatory amino acids glutamine, leucine, and arginine to starved cells reactivated MTORC1 and blocked the autophagy. Thus, the 3 main regulatory amino acids are sufficient to control autophagosome assembly.

When considering autophagy and the different nutrients, our data highlighted the fact that time was a key variable. The clearest differences between amino acid starvation and glucose starvation were observed during the immediate rapid autophagy response (e.g., up to 2 h). Therefore, amino acid-dependent MTORC1-ULK1 signaling primarily serves to promote high rates of LC3 conversion and autophagosome assembly. During prolonged starvation experiments, we observed other ULK1/2-independent pathways, which we interpret to function at lower rates, becoming apparent in longer time frames. Cellular LC3 levels are effectively cleared upon prolonged amino acid starvation, likely reflecting sustained activation of lysosomal function. Basal autophagosome formation was seen to be ULK1/2 independent after prolonged blocking of flux (i.e., Baf A1). Serum starvation also produced mild effects on autophagosome formation, MTORC1 activity, and lysosomal acidification. We suggest that considerations of time frame and serum levels may explain some of the observations of glucose starvation-induced, noncanonical, ULK1/2-independent autophagy (5, 14, 17, 19). Overall, amino acid and glucose starvation produced clearly different effects across many cell types.

### AMPK and glucose starvation signals can dominate and block autophagy.

Interestingly, while amino acid starvation stimulated autophagy, further removal of glucose under amino acid-free conditions blocked autophagosome formation. Previously, it had been proposed that autophagy initiation in both mammalian and yeast cells requires threshold levels of cellular energy (20, 23, 50). Glucose starvation in our experiments clearly reduced the energy charge in cells, as reflected by AMPK activation and phosphorylation of the ULK1-Ser555 site. Altogether, the highest levels of autophagy were associated with amino acid deprivation and hypophosphorylation of ULK1 at Ser555 and Ser757. As such, canonical rapid autophagy seems to be driven by suppression of MTORC1 in association with low AMPK activity.

We were next able to show that activation of AMPK by using a drug was able to suppress the otherwise positive signal of amino acid starvation for autophagosome formation. This brake mechanism on autophagy appears to require the set of 4 conserved AMPK phosphorylation sites on ULK1 (including Ser555), which had been previously validated functionally (9). Therefore, high levels of AMPK-mediated phosphorylation on these sites may serve to inhibit ULK1. However, the relationship is not binary, since 4SA mutation of these sites also prevented ULK1 from promoting normal autophagy. One possibility is that transient or subthreshold levels of AMPK phosphorylation on these sites are needed for proper dynamic regulation of ULK1. Sustained high levels of modification may serve as a signal to block ULK1. Alternatively, one of the sites in this set may function as the brake, but the 4SA substitutions together may inhibit other positive roles, although this will require more mapping. Indeed, one of the sites in 4SA is Ser637 (638 in humans), which is also coordinately regulated by MTORC1 and PP2A in response to nutrients (10, 51). ULK1-Ser555 phosphorylation may provide a switch from canonical autophagy to mitophagy-specific pathways following AMPK activation (8, 9).

### Amino acid and glucose differentially control the lysosome.

We consistently saw that amino acid starvation, especially when prolonged, generated LC3 conversion, LC3 breakdown, and MTORC1 reactivation. In contrast, glucose starvation led to only slow accumulation of lipidated LC3-II and never restimulated MTORC1, which we interpreted to reflect overall lysosomal suppression. This model suggests further considerations that may explain the LC3 accumulation observed in other examples of glucose starvation (5, 14, 17, 19).

Autophagy flux depends on fusion with the lysosome to enable content degradation. Lysosomal function can be upregulated following gene expression reprogramming and organelle biogenesis driven by TFEB family transcription factors (52). Alternatively, existing lysosomes can be activated by promoting lumenal acidification, which has been reported to occur via both MTORC1-dependent and -independent mechanisms (41, 53). Here, we found that serum starvation promoted some acidification but that the strongest lysosomal activation occurred when both serum and amino acids were withdrawn. This lysosomal response occurred rapidly and independently of the ATG5 and ULK1/2 autophagy pathways. Glucose starvation did not stimulate lysosomal acidification, consistent with the other data suggesting low autophagic flux. Looking at nutrient combinations, removal of glucose prevented amino acid and serum starvation from promoting lysosome acidification. Furthermore, activation of AMPK was sufficient to suppress lysosomal activation. Therefore, AMPK has ULK1-dependent pathways to control early autophagy steps and distinct pathways to control the lysosome.

Lysosomal acidification is driven by vATPase, which, interestingly, displays nutrient-dependent assembly of its V0 and V1 domains (54). Amino acid starvation has been shown to promote vATPase assembly, although the role of MTORC1 in this mechanism remains controversial (41, 53, 55). Conversely, vATPase assembly has been shown in yeast and mammals to require glucose (56, 57). Here, we identified an additional pathway involving AMPK activity to suppress lysosomal function. Therefore, vATPase may be blocked via multiple mechanisms to produce a reduction in autophagy-lysosomal flux upon glucose starvation.

In conclusion, our studies provide an integrated view of how serum, amino acid and glucose independently control early and late stages of autophagy. For both autophagosome formation and lysosomal acidification, amino acid starvation provided the strongest activating signal. Surprisingly, both early and late stages of autophagy were not activated by glucose starvation, and moreover, glucose starvation had overall inhibitory effects on both pathways. The inhibitory effects of glucose starvation were determined to take place via distinct AMPK-dependent mechanisms on autophagy initiation and lysosomal activity. The mechanisms characterized here may help coordinate the physiological homeostasis of amino acids, glucose, and autophagy, as seen in neonatal mice (26). Our findings here also illustrate that starvation of different nutrients cannot be generalized to activate autophagy.

## MATERIALS AND METHODS

### Cell culture and treatments.

ULK1/2 DKO MEF (11), WT MEF expressing GFP-DFCP1 (ZFYVE1) (11), and FIP200 KO (58) and ATG5 KO MEF (and matched WT MEF) (59) have been described previously. HEK293A cells were maintained as previously described (31). MEF and HEK293A, HeLa, 4T1, SKOV3, OVCAR, B16-F0, and A431 cells were all maintained in DMEM with 4.5 g/liter glucose (Lonza; BE12-614F) supplemented with 10% fetal bovine serum (FBS) (Labtech; FCS-SA), 4 mM l-glutamine (Lonza; BE17-605E), and 100 U/ml penicillin-streptomycin (Lonza; DE17-602E) (full-nutrient medium). MCF7 cells were cultured in full-nutrient medium supplemented with 0.015 mg/ml insulin.

Where indicated, WT and ULK1/2 DKO MEF were transiently transfected with tandem-tagged mRFP-enhanced GFP (EGFP)-LC3 reporter (28). Alternatively, HEK293A and A431 cell lines stably expressing pBABE-puro mCherry-EGFP-LC3B (Addgene plasmid 22418) were generated (60). A HEK293A stable cell line expressing LAMP1-mRFP-FLAG (Addgene plasmid 34611) was generated (61). MEF with stable knockdown of beclin1 were generated using the pLKO.1 construct for a mouse BECN1 clone (Broad Institute Genetic Perturbation Platform construct number TRCN0000087290). In reconstitution experiments, ULK1/2 DKO MEF were stably transduced using pLPC puro-Myc-ULK1 WT or 4SA (S467A, S555A, T574A, and S637A) (subcloned from Addgene plasmids 27626 and 27628) (9).

Cells were washed once with phosphate-buffered saline (PBS) and exchanged into starvation medium. For amino acid (and serum) starvation, we used EBSS (Sigma; E2888). For glucose (and serum) starvation, we used glucose-free DMEM containing 4 mM l-glutamine (Thermo Fisher; 11966-025). For serum starvation, we used full-nutrient DMEM as described above but lacking FBS. For amino acid and glucose (double) starvation, we used PBS (Lonza; BE17-513F) supplemented with 0.22% sodium bicarbonate (Sigma; S8761) and phenol red. To study adding back glucose, we used PBS containing 1 g/liter glucose (Lonza; 04-479Q) supplemented with 0.22% sodium bicarbonate and phenol red. Where indicated, media were supplemented with dialyzed FBS (Sigma; F0392) or with 4 mM glutamine (Lonza; BE17-605E), 0.8 mM leucine (Sigma; L8912), and 0.4 mM arginine (Sigma; A8094). Some experiments used 10 nM bafilomycin A1 (Tocris Bioscience) or 25 μM chloroquine (Sigma) to inhibit the lysosome. AMPK was activated using 50 μM A769662 (Tocris Bioscience). MRT68921 (35) was a kind gift from B. Saxty (LifeArc, formerly MRC Technology).

### Immunoblot analysis.

Cell lysates were prepared as described previously (11) and analyzed using 4% to 12% or 10% NuPAGE gels resolved in MES (morpholineethanesulfonic acid) running buffer (Thermo Fisher Scientific). Membranes were stained using the following antibodies: LC3B, clone 5F10 (Nanotools; 0231-100); p62/SQSTM1 (BD Bioscience; 610832); phospho-S6 Ser240/Ser244 (Cell Signaling; 2215); total S6 (54D2) mouse monoclonal antibody (MAb) (Cell Signaling; 2317); phospho-ACC Ser79 (Cell Signaling; 3661); total ACC (Cell Signaling; 3662); phospho-ULK1 Ser757 (Cell Signaling; 6888); phospho-ULK1 Ser555-D1H4 (Cell Signaling; 5869); phospho-ULK1 Ser317-D2B6Y (Cell Signaling; 12753); total ULK1-D8H5 (Cell Signaling; 8054); and actin, Ab-5 (BD Bioscience; 612656). Detection was via anti-mouse or anti-rabbit Dylight-coupled secondary antibodies and LiCor Odyssey infrared scanning.

### Microscopy.

After treatments, cells were fixed and stained using the following antibodies: anti-human p62/SQSTM1 (BD Bioscience; 610832), anti-mouse p62/SQSTM1 guinea pig polyclonal (Progen; GP62-C), LC3B (Cell Signaling; 2775), MTOR-7C10 (Cell Signaling; 2983), ATG13-E1Y9V (Cell Signaling; 13468), and ULK1-D8H5. Cell images were captured by confocal microscopy (Leica; TCS SP5; HCX PL APO CS-63×-1.4-numerical-aperture [NA] objective and HyD GaAsP detection). The number of puncta per cell was determined from confocal scans or directly by epifluorescent imaging, depending on the stain. To detect lysosomal acidification, cells were treated as indicated with 50 nM Lysotracker Red DND-99 (Thermo Fisher; L7528) added during the final 30 min of incubation. The cells were fixed with paraformaldehyde, stored overnight, and imaged by confocal microscopy. The staining intensity was quantified from cytoplasmic regions of interest from multiple cells per field from multiple fields per sample. Quantification of autophagosome puncta and Lysotracker staining were representative of multiple experiments, as detailed in the figure legends.

### Transcript analysis.

RNA was extracted from cells using Isolate II RNA minicolumns (Bioline). Expression analysis was carried out using the Luna one-step reverse transcription-quantitative PCR (RT-qPCR) kit and the following primers: Hs *LC3B*, forward, ACG CAT TTG CCA TCA CAG TTG, and reverse, TCT CTT AGG AGT CAG GGA CCT TCA G; Hs *p62/SQSTM1*, forward, CCG TGA AGG CCT ACC TTC TG, and reverse, TCC TCG TCA CTG GAA AAG GC; Hs *GAPDH*, forward, CTA TAA ATT GAG CCC GCA GCC, and reverse, ACC AAA TCC GTT GAC TCC GA. The gene fold change normalized to *GAPDH* was calculated using ΔΔ*C_T_* analysis.

### Statistics.

Quantitative data were managed using GraphPad Prism and Origin Pro and analyzed using an unpaired *t* test (for 2-way comparisons) or one-way analysis of variance (ANOVA) with the Tukey posttest (for multiple comparisons), as appropriate.
